# Innate immune sensing of alphavirus chikungunya: balancing antiviral defense and pathogenesis

**DOI:** 10.3389/fimmu.2026.1810208

**Published:** 2026-04-29

**Authors:** Juliane Santos de França da Silva, Célio Valdevino Ferreira Junior, Livian Maria Silva dos Santos, Valter Feirreira de Andrade-Neto, Paulo Marcos da Matta Guedes, Juliana Navarro Ueda Yaochite, Rafael Freitas De Oliveira França, Ramayana Morais de Medeiros Brito, Manuela Sales Lima Nascimento

**Affiliations:** 1 Department of Microbiology and Parasitology, Biosciences Center, Federal University of Rio Grande do Norte, Natal, Rio Grande Do Norte, Brazil; 2 Faculty of Pharmacy, Dentistry & Nursing, Federal University of Ceará, Fortaleza, Ceará, Brazil; 3 Laboratory of Translational Medicine. Oswaldo Cruz Foundtion (FIOCRUZ), Ribeirão Preto, Brazil; 4 Division of Biomedical Science and Biochemistry, Research School of Biology, The Australian National University, Canberra, ACT, Australia

**Keywords:** chikungunya virus, immunopathogenesis, innate immunity, pattern recognition receptors, type I interferons

## Abstract

Alphavirus chikungunya (CHIKV) is an arthritogenic virus whose innate recognition is primarily driven by pathogen associated molecular patterns (PAMPs) or damage associated molecular patterns (DAMPs), sensed by a variety of pattern recognition receptors (PRRs) such as Toll-like receptors (TLR), and cytosolic RIG-like receptors. CHIKV infection elicits a multifaceted interplay between viral replication strategies and host innate immune recognition. Among these pathways, TLR-mediated sensing emerges as a central axis of antiviral defense, orchestrating type I interferon responses and inflammatory cascades that determine the balance between viral clearance and immunopathology. In parallel, inflammasomes such as NLRP3 amplify the IL-1β/IL-18 axis, being a major contributing factor for the establishment of chronic joint inflammation. The transition from self-limited rapid resolving infection to chronic disease is largely determined by the cytokine and chemokine milieu. It is known that acute infection is characterized by high levels of IL-6, TNF-α, and IL-1β, which drive fever, myalgia, and joint inflammation; and chemokines such as CCL2 (MCP-1) were shown to recruit monocytes and macrophages to inflamed joints, whereas CXCL9/10 (MIG/IP-10) enhance T-cell trafficking, contributing to viral clearance but also sustaining tissue inflammation. In this review, we aim to consolidate the current knowledge on the molecular pathways that sense CHIKV infection and trigger the antiviral innate response that can act as both protective and pathogenic. By integrating viral evasion strategies and host factors, we provide a comprehensive framework for understanding innate immunity in CHIKV infection, its implications for therapeutic design, and important gaps that can guide future studies.

## 1 Introduction

Alphavirus chikungunya (CHIKV) is an Old World Alphavirus well known for causing arthritogenic disease with high morbidity (1). Transmitted by *Aedes aegypti* and *A. albopictus* mosquitoes, it was first identified in Tanzania in 1952 (2, 3). It re-emerged in 2004 as a global public health threat, spreading across the Indian Ocean and the Caribbean, subsequently reaching the Americas (4). Currently, CHIKV is widely distributed and recognized as the arthritogenic arbovirus with the broadest global range, responsible for large-scale outbreaks (5). Approximately 1.5% of the susceptible population is estimated to be infected by CHIKV annually, resulting in about 17.8 million symptomatic cases and 3500 deaths per year (6).

Chikungunya disease (CHIKD) presents with fever, headache, fatigue, myalgia, rash, and severe arthralgia (7). Approximately 56% of patients fully recover, while the remainder progress to the chronic phase, experiencing persistent arthralgia for months or years, often resulting in polyarthralgia or debilitating polyarthritis (7, 8). Clinical manifestations involving neurological, cardiovascular, pulmonary, and ophthalmological complications have also been reported (9, 10), with severe cases resulting in fatal outcomes more frequently observed in neonates and the elderly (11–13).

It is well established that innate immunity plays a central role in controlling CHIKV infection and in shaping the disease outcomes (14). The initial response is triggered by the recognition of viral components by Pattern Recognition Receptors (PRRs), a diverse family of sensors that are able to recognize pathogen-associated molecular patterns (PAMPs) or damage-associated molecular patterns (DAMPs). Among these receptors, Toll-like receptors (TLRs), RIG-I-like receptors (RLRs), and inflammasomes are expressed in cells at the frontline of CHIKV infection, including macrophages, dendritic cells, and fibroblasts (15–17). They have been most prominently implicated in the recognition of PAMPs, such as viral RNA, or DAMPs, such as mitochondrial DNA, and activate downstream signaling pathways that trigger the production of inflammatory cytokines, chemokines, and other antiviral molecules. Together, all these innate immunity mediators induced in response to an initial viral recognition, not only suppresses viral replication but also act in the recruitment and activation of additional immune effectors, orchestrating a dynamic and complex host antiviral defense (18–20). Polymorphisms in genes encoding transmembrane proteins, enzymes, soluble mediators, and pattern recognition receptors involved in the immune response to CHIKV infection may modulate CHIKD pathogenesis and progression (21).

Although essential for limiting viral spread, the innate immune response can also act as a double-edged sword in the context of CHIKV infection. When excessively prolonged or dysregulated, these same pathways drive persistent inflammation, contributing to disease chronicity and development of long-lasting arthralgia. Studies have shown that sustained inflammation has been closely related to the transition from acute febrile illness to debilitating chronic manifestations, underscoring the delicate balance between protective immunity and pathogenesis during CHIKV infection (22–24). Therefore, understanding how innate immune pathways contribute both to viral clearance and long-term sequelae is of major necessity to unravel the immunopathogenesis of CHIKD. In this review, we provide a comprehensive overview of key molecular mechanisms underlying the innate immunity in CHIKV infection. By integrating insights into viral sensing, cytokine and chemokine signaling, and immune-driven pathology, we aim to shed light on the dual role of innate immunity during CHIKV infection, highlight potential avenues for therapeutic interventions, and identify critical knowledge gaps that will guide future research directions in the field.

## 2 CHIKV replication cycle, major PAMPs and DAMPs inducers

As an enveloped, positive-sense, single-stranded RNA (ssRNA) virus of approximately 11.8 kb, CHIKV genome is 5′-capped and 3′-polyadenylated and encodes a nonstructural polyprotein (P1234), which is further cleaved into four nonstructural proteins (nsPs 1-4), and into structural proteins named: C (capsid), the envelope glycoproteins E1, E2, and E3, as well as the 6K protein and its translational frameshift product, TF (25–27).

**Figure 1:**
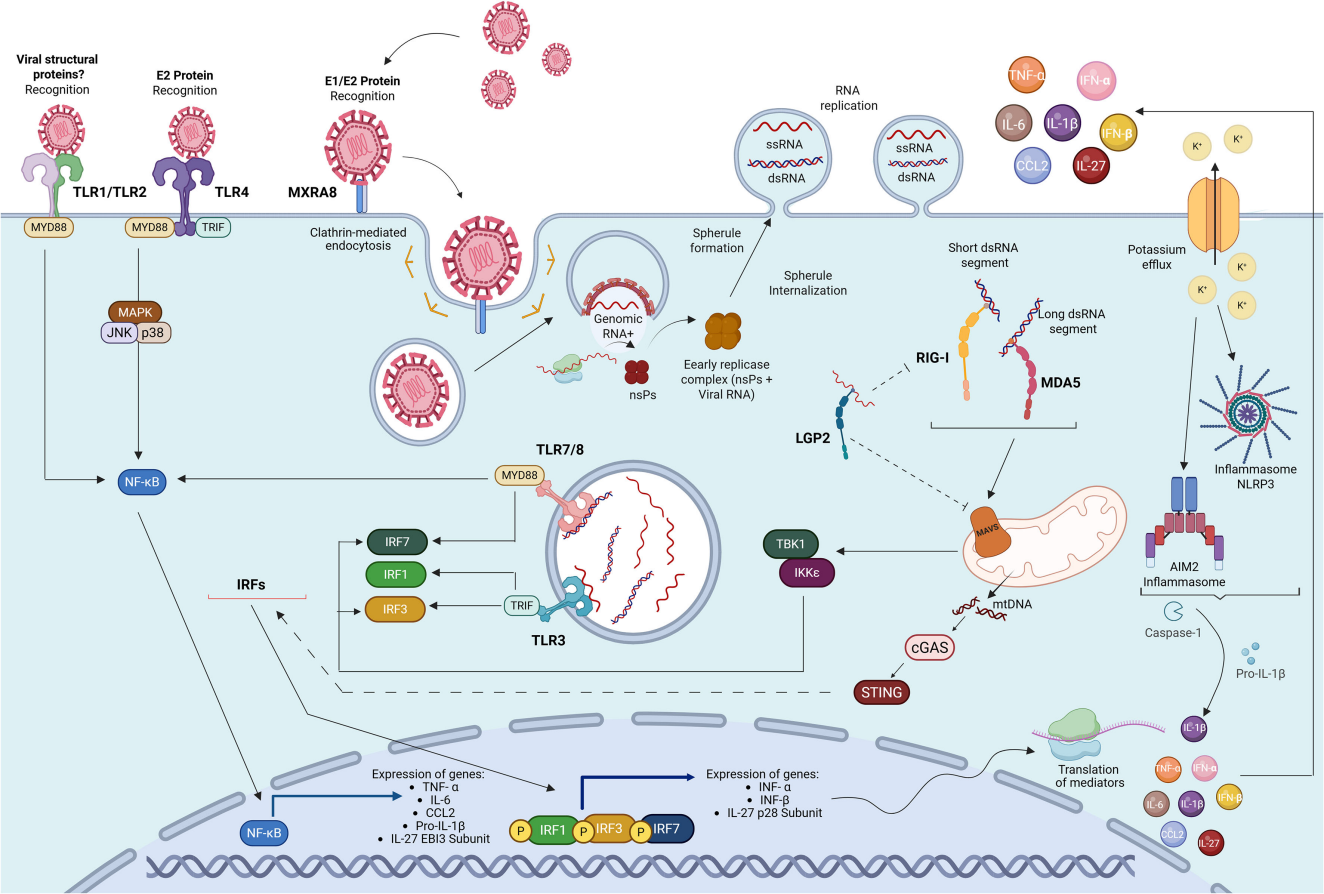
Overview of innate immunity signaling pathways engaged during CHIKV infection. Viral PAMPs are sensed at the cell surface by PRRs such as TLR4 and TLR1/2, leading to NF-κB activation and the induction of inflammatory gene expression. CHIKV entry is mediated by clathrin-mediated endocytosis, followed by fusion of the viral envelope with the endosomal membrane, releasing the positive-strand genomic RNA into the cytoplasm. Translation of the non-structural proteins gives rise to the early replicase complex, which associates with the cell membrane and induces the formation of spherule-shaped replication organelles. Viral RNA may access endosomal TLR3 and TLR7/8 through alternative pathways, such as endocytosis of virions, uptake of infected cell material, or autophagy-mediated delivery to endolysosomes (not illustrated), potentially enabling the recognition of dsRNA and ssRNA and the activation of IRF- and NF-κB–dependent signaling pathways that drive inflammatory and antiviral responses. In the cytosol, dsRNA intermediates are also recognized by RIG-I and MDA5, signaling via MAVS to promote IFN-I production, while LGP2 binds CHIKV RNA and may exert modulatory functions. Cytoplasmic accumulation of mtDNA acts as a DAMP, triggering the cGAS–STING pathway and further IRF activation. In parallel, ionic imbalance and potassium efflux activate NLRP3 and AIM2 inflammasomes, leading to caspase-1 activation and IL-1β secretion. Adapted from (36, 203). Image created in https://BioRender.com. Created in BioRender. de França da Silva, J. S. (2026) https://BioRender.com/yh5nkpu.

The viral entry is initiated by E2-mediated attachment to host cells, facilitated by interactions with heparan-sulfate and related glycosaminoglycans in the target cell surface, which enhance attachment through electrostatic interactions and influence cell tropism and infectivity (28). The matrix remodeling-associated protein 8 (MXRA8, also known as limitrin, DICAM, or ASP3) functions as a high-affinity receptor for multiple arthritogenic alphaviruses, including CHIKV, acting by stabilizing virion binding with both E2 and E1 proteins and promoting viral uptake (29, 30). Following attachment, the virus enters through clathrin-mediated endocytosis. Acidification within endosomes triggers conformational changes in the E1 glycoprotein, promoting viral–endosomal membrane fusion (25, 31, 32), thereby releasing the viral RNA in the cytoplasm (**Figure 1**). The P123 and P1234 polyproteins are translated, and subsequent cleavage of P1234 releases the mature nsP4. P123 and nsP4 form an early replicase complex with the viral RNA that associates with host factors at the plasma membrane, reshaping it into replication organelles termed spherules. Within these structures, negative-strand RNA intermediates are synthesized, later switching to the transcription of new positive-stranded genomic RNA and subgenomic RNA-encoding structural proteins (33–35). Structural proteins encoded by subgenomic RNA are processed in the endoplasmic reticulum–Golgi pathway and then transported to the plasma membrane. Concurrently, capsid proteins package the viral genome into nucleocapsids, which assemble with envelope glycoproteins at the plasma membrane to drive budding and virion release (26).

Spherules shield viral double-stranded RNA (dsRNA) intermediates from cytosolic innate sensors (36). Although CHIKV replication compartments are formed at the plasma membrane, direct access of viral RNA to endosomal TLRs is unlikely due to spatial compartmentalization. Instead, viral RNA may become accessible to endosomal TLRs through alternative pathways, such as endocytosis of virions, uptake of viral material from infected cells, as well as autophagy-mediated delivery of cytoplasmic RNA to endolysosomes. However, these mechanisms have not yet been experimentally demonstrated for CHIKV, highlighting an important gap in our understanding of how endosomal TLRs are engaged during infection. Similar processes have been described for other RNA viruses, supporting their biological plausibility (37, 38). Thus, multiple CHIKV components may function as PAMPs, triggering innate immune recognition and activation, such as viral RNAs serving as potent ligands for RIG-I, MDA5, TLR3, TLR7 and TLR8 (**Figure 1**). Moreover, host cell’s C-type lectin receptors may also recognize PAMPs from CHIKV, as DC-SIGN has been suggested to interact with E1 glycoprotein (39), although confirmation is required.

The CHIKV 6K/TF protein has been characterized as a functional viroporin, forming ion channels that disrupt host cell membranes, promoting vesicle fusion, and facilitating viral budding (40). Such membrane perturbations may secondarily contribute to innate immune activation via DAMPs. In contrast, the capsid protein (C) primarily functions in nucleocapsid formation and RNA packaging (41), with no direct evidence supporting its role in membrane disruption or innate sensing. Furthermore, there is no evidence that CHIKV nonstructural proteins (nsPs) can act as PAMPs or as DAMPs inducers. Instead, it was shown that these proteins function as essential enzymatic and regulatory elements of viral replication (42), acting as antagonists of innate immunity, helping the virus to evade detection (43–45).

The pathogen-host interactions in CHIKV infection involve complex mechanisms that delineate the host resistance or susceptibility. Dissecting how CHIKV activates and modulates PRRs is therefore critical to understand the protective and pathogenic dimensions of the innate immune response, particularly in relation to arthralgia and chronic disease outcomes.

## 3 TLR-mediated sensing of CHIKV, downstream adaptors and cytokine production

Toll-like receptors (TLRs) were the first family of innate immune receptors to be characterized (46) and are expressed by a wide range of immune cells, including DCs, macrophages, neutrophils, B cells. Non-immune cells such as epithelial cells and fibroblasts also express TLRs and contribute actively to innate immune responses through the recognition of PAMPs. In humans, ten functional TLRs (TLR1–TLR10) have been identified, whereas mice express twelve (TLR1–TLR9 and TLR11–TLR13); with TLR1–TLR9 being conserved across both species. Mouse TLR10 is shown to be a non−functional receptor, and TLR11–TLR13 are absent from the human genome (47, 48). Those receptors can be located in the cell membrane (TLR1, TLR2, TLR4, TLR5, TLR6, and TLR10), recognizing PAMPs from extracellular environment, such as lipoproteins and glycoproteins, or within intracellular compartments such as endosomes and endolysosomes (TLR3, TLR7, TLR8, and TLR9), where they can detect internalized microbial components, such as viral nucleic acids. Notably, several TLRs play a critical role in CHIKV defense by sensing viral RNA or proteins, signaling through TRIF or MYD88 adaptor molecules and inducing key cytokines (**Table 1**).

**Table 1:** TLRs in CHIKV infection ranked by functional relevance.

TLR	Expressing cell populations	Cell compartment	Ligand	Pathway	Function in CHIKV infection	Reference
TLR7	Monocytes Neutrophils	Endosomes	Viral ssRNA	MyD88	Strong inducer of IFN-α; limits early viremia Required for NETosis via ROS	(62, 204)
TLR3	Epithelial cells, Fibroblasts MDM Hematopoietic cells (B cells)	Endosomes Endosomes Endosomes	Viral dsRNA, Poli I:C, AV-C Viral dsRNA Viral dsRNA	TRIF/IRF3/IRF1 TRIF/IRF1 TRIF	IFN-β induction and CHIKV replication suppression Induces IL-27p28 Modulates early CHIKV‐specific IgG response	(55–57, 73)
TLR8	Monocytes, macrophages, mDCs	Endosomes	Viral ssRNA	MyD88/IRF7, NF-κB	Induces IFN-α, IL-6, TNF-α	(62)
TLR2	MDM Astrocytes Monocytes	Plasma membrane Plasma membrane Plasma membrane	Viral structural proteins? Viral structural proteins? Viral structural proteins?	MyD88/NF-κB	Involved in IL-27 production (EBI3 subunit) Astrogliosis Early production of TNF-α, IL-6, and IL-1β	(62, 72, 73)
TLR1	MDM	Plasma membrane	Viral structural proteins?	MyD88/NF-κB	Function with TLR2 to induce IL-27 production (EBI3 subunit)	(73)
TLR4	Macrophage MDM	Plasma membrane Plasma membrane	Envelope protein E2 Bacterial LPS	MyD88 and/or TRIF/NF-κB/MAPK MyD88 and TRIF	Induction of inflammation: TNF-a, IL-6 and CCL2 Induction of IL-27, attenuation of CHIKV replication	(73, 76)
TLR9	Fibroblasts	Endosomes	CpG DNA Viral ssRNA?	MyD88/IRF7	Protection against CHIKV-induced encephalitis and reduces CHIKV infection	(77, 78)
TLR5	Not studied in CHIKV infection					
TLR6	Not studied in CHIKV infection					
TLR10	Not studied in CHIKV infection					

The cells and the ligands presented in this table are restricted to ones described in the context of chikungunya. TLR, Toll-Like Receptor; MDM, monocyte-derived macrophage; AV-C,1-(2-fluorophenyl)-2-(5-isopropyl-1,3,4-thiadiazol-2-yl)-1,2-dihydrochromeno[2,3-c]pyrrole-3,9-dione; MyD88, Myeloid differentiation primary response protein 88; ssRNA, single-stranded RNA; dsRNA, double-stranded RNA; LPS, lipopolysaccharide; TRIF, TIR-domain-containing adapter-inducing interferon; IL, interleukin; IRF, Interferon Regulatory Factor.

TLR3 is specialized in the recognition of double-stranded RNA (dsRNA), a replicative intermediate commonly generated during the life cycle of RNA viruses, including CHIKV. Upon activation, TLR3 signals through adaptor protein TRIF, leading to the induction of NF-κB complex and interferon regulatory factors (IRFs). This signaling cascade culminates in the production of type I interferons and proinflammatory cytokines (49, 50). TLR3 expression has been documented in myeloid DCs, macrophages, mast cells, fibroblasts, epithelial cells and other non-hematopoietic cells (51–53), which represent important early targets of CHIKV infection (20, 54).

Compelling evidence from both *in vitro* and *in vivo* studies supports a critical role for TLR3 in host defense against CHIKV. In epithelial cell lines such as BEAS-2B, activation of TLR3 significantly suppresses CHIKV replication, indicating its antiviral potential at barrier sites (55). Similarly, both human and murine fibroblasts lacking TLR3 or harboring defects in TLR3 signaling display increased susceptibility to CHIKV infection (56). Importantly, the TRIF-dependent signaling cascade downstream of TLR3, which culminates in IRF3 activation and subsequent production of type I interferons (IFN-I), has been shown to restrict replication not only of CHIKV, but also of Zika virus (ZIKV) and dengue virus (DENV) in human fibroblasts, reinforcing the broad antiviral potential of the TLR3–TRIF axis in non-hematopoietic cells (57).

*In vivo*, experiments using bone marrow chimeric mice demonstrated that TLR3-expressing hematopoietic cells are the primary populations responsible for effective CHIKV clearance. Moreover, TLR3 deficiency in mice leads to higher CHIKV replication and exacerbated inflammation. These effects are accompanied by reduced production of type I interferons and impaired neutralizing antibody responses, underscoring the importance of TLR3 in orchestrating both innate and adaptive antiviral immune responses (56). Additionally, translational studies in peripheral blood samples from patients with acute CHIKD have reported increased transcription of TLR3 and TRIF, which correlate with increased expression of IL-6 and IFN-α mRNA, critical cytokines to inflammatory and antiviral responses, respectively (58).

In patients, the TLR3 single nucleotide polymorphism (SNP) rs6552950 was associated with increased disease severity (56), and the A allele of TLR3 (rs3775291) implicates individuals with a functionally impaired receptor (AA genotype) also being more susceptible to CHIKD (59). These findings suggest that genetic variation in TLR3 may modulate susceptibility to CHIKV-induced immunopathology. Finally, TLR3 is involved not only in peripheral antiviral defense but also in neuroimmune responses (60), as in murine models of CHIKV neuroinvasion, infection leads to significant upregulation of TLR3 expression in the brain, along with increased transcription of key downstream signaling components, including TICAM-1, TRAF6, IRF1, IRF3, and IRF7 (60).

Other endosomal receptors important for viral PAMPs detection are TLR7 and TLR8, activated by single-stranded RNA (ssRNA) from viruses. TLR7 is predominantly expressed in plasmacytoid dendritic cells (pDC) and B cells, while TLR8 is more frequently found in monocytes, macrophages, myeloid DCs, and neutrophils. Both receptors signal through the MyD88 adaptor molecule, leading to the activation of IRFs and also NF-κB. These pathways drive the production of several cytokines including IFN-I, which have been consistently reported during the acute phase of CHIKV infection, as early as the first week of symptoms onset (58, 61).

Although the expression of TLR7 and TLR8 is not upregulated in PBMCs during acute CHIKD in humans (58), it has been shown that CHIKV challenge led to increased TLR7 and TLR8 transcript levels in human monocytes in *in vitro* assays (62), although whether this reflects enhanced receptor activation in response to the infection remains unclear. Experimentally, mice lacking TLR7, or with MyD88-mediated signaling disrupted, a key adaptor downstream of TLR7/8 and other TLRs, display elevated viral loads at early stages of CHIKV infection, highlighting the key role of this pathway for initial viral control. The absence of MyD88-mediated signaling has also been associated with increased viral replication specifically in Ly6C^hi^ inflammatory monocytes, a key cell population in CHIKV pathogenesis (63). The role of TLR7 in limiting viral spread via innate effector mechanisms has been experimentally demonstrated in murine neutrophils infected with CHIKV, where the virus was able to induce NETosis via a pathway dependent on TLR7 signaling and the generation of reactive oxygen species (ROS) (64).

Single nucleotide polymorphisms (SNPs) in TLR7 and TLR8 have been associated with altered susceptibility and clinical outcomes in CHIKV-infected individuals. In a Brazilian cohort, the G allele of the TLR7 variant (rs3853839 G/C) and the G allele of TLR8 (rs3764879 G/C) were significantly associated with protection against symptomatic infection. Notably, individuals carrying the TLR8 rs3764879 G allele were more likely to remain asymptomatic following exposure to CHIKV, suggesting a protective role for this genetic trait (65). In an Indian cohort, the polymorphisms in TLR7 (rs179010 C/C in male, and rs5741880 G/T in female) and TLR8 (rs3764880, in linked disequilibrium with rs3764879) were correlated with increased susceptibility to CHIKV infection and more severe clinical manifestations, such as fever, joint pain and rash (66). Another study reported that individuals from Eastern India carrying the CC genotypes of rs179008 and rs3853839 in TLR7, as well as rs3764879 and rs5744080 in TLR8, showed increased susceptibility to dengue virus (DENV)–CHIKV co-infection. In contrast, the GC genotype of rs3853839, the GC genotype of rs3764879, and the AG genotype of rs3764880 (TLR8) were associated with a higher susceptibility to CHIKV mono-infection (67). Collectively, these studies highlight that TLR7 and TLR8 polymorphisms may modulate immune activation and inflammation during CHIKV infection, contributing to interindividual variability in disease progression and clinical presentation.

Nevertheless, microbiota-derived signals are also critical modulators of the TLR7-MyD88 pathway. Germ-free or antibiotic-treated mice exhibited impaired TLR7-MyD88 signaling and increased CHIKV viremia, highlighting a complex interaction between host microbiota and antiviral immunity (63). Importantly, disruption of pDC-derived type I interferon production, caused by alterations in the microbiota, has also been linked to increased susceptibility of Ly6Chi monocytes to infection by both CHIKV and Mayaro virus (MAYV) (63), further underscoring the contribution of TLR7-mediated signaling in early antiviral defenses.

Toll-like receptor 2 can form a cell-surface heterodimer with TLR1, TLR4, TLR6 or TLR10 (68–70), engaging lipopeptides and other PAMPs (71). TLR2 participated in sensing CHIKV structural components promoting activation of human monocytes from the early stages of infection. The CHIKV upregulates TLR2 expression in those cells and it is associated with early production of TNF-α, IL-6, and IL-1β (62). Furthermore, CHIKV infection in nonhuman primates leads to sustained upregulation of TLR2 in astrocytes, even after the virus has been cleared from the circulation. This was associated with astrogliosis, suggesting that TLR2-driven innate immune activation may contribute to neuroinflammation (72).

Recent work by Valdés-López et al. (2022) demonstrated that CHIKV infection of human monocyte-derived macrophages (MDM) induces interleukin-27 (IL-27) production through a dual-pathway mechanism involving TLR1/2–MyD88 and TLR3–TRIF signaling. In this model, early activation of TLR1/2 initiates NF-κB–mediated transcription of the EBI3 subunit of IL-27, while subsequent TLR3 engagement induces IRF1-dependent expression of IL-27p28. Blockade of either TLR1/2 or MyD88 impairs IL-27 secretion and downstream induction of ISGs (73). IL-27 triggers JAK-STAT signaling and promotes pro-inflammatory and antiviral response, in an IFN-independent manner (74). These results highlight the interplay between endosomal and surface TLRs in regulating IL-27 production and expand our understanding of how macrophages integrate pathogen-sensing signals to modulate innate immunity during arboviral infections.

TLR4 activation also contributes to macrophage responses during CHIKV infection. TLR4 stimulation with monophosphoryl lipid A (MPLA) as an adjuvant for an inactivated CHIKV vaccine induces higher levels of neutralizing antibodies compared to non-adjuvanted formulations (75), highlighting TLR4 as a potential target for vaccine design. Moreover, TLR4 responds to viral proteins and DAMPs generated during infection. A landmark study by Mahish et al. (2023) demonstrated that TLR4 acts as a receptor for the CHIKV envelope protein E2 in macrophages, triggering a pro-inflammatory cascade through NF-κB and MAPK signaling, characterized by elevated TNF-α, IL-6, and CCL2 production (76). Finally, pharmacological inhibition of TLR4 reduces viral copy numbers as well as CHIKV E2 protein levels (76), supporting a role for this pathway in CHIKV pathogenesis.

Although TLR9 is classically associated with DNA sensing inside the endosomes, evidence suggests it may contribute to antiviral defenses against RNA viruses such as CHIKV. Activation of TLR9 by CpG oligonucleotides protected mice from CHIKV-induced encephalitis. This protection was mediated by enhanced NK cell activity and increased production of types I and II IFN and TNF-α (77), highlighting a potent immunomodulatory and neuroprotective role of TLR9 signaling in this model. The use of innate immune modulators offers a promising approach to potentiate host antiviral defenses and mitigate neuropathological outcomes associated with neurotropic CHIKV infection. Complementarily, interferon-stimulated genes (ISGs) IFITM1, IFITM2, and IFITM3 overexpression causes upregulation of TLR3, TLR7, TLR8, and TLR9 and also inhibit CHIKV infection in human skin fibroblasts (78).

While several TLRs have clearly defined roles in CHIKV sensing and immune activation, other members of the TLR family remain underexplored in the context of this virus. There is no direct evidence linking TLR5, TLR6, or TLR10 to CHIKV infection, but their potential roles cannot be excluded. TLR6 forms heterodimers with TLR2 and has been implicated in the recognition of viral components in other arboviruses such as Dengue virus (79). TLR5, best known for recognizing bacterial flagellin, may influence antiviral immunity indirectly by shaping the innate immune environment, especially in the presence of microbiota-derived signals. And last but not least, TLR10, expressed in human myeloid cells, has been shown to exhibit, when overexpressed, an inhibitory activity against TLR2-mediated signaling (80). The absence of data regarding these TLR5, 6, and 10 in CHIKV infection represents an important gap in the current literature and points towards a new avenue for future investigation. Delving deeper in the understanding of non-canonical TLR pathways role in shaping antiviral immunity could provide insights into host factors that underlie variabilities in disease severity, chronic inflammation, and viral persistence, thereby fine-tuning innate immune responses during CHIKV infection. Addressing these gaps could help redefine the current models of CHIKV-host interactions and reveal novel regulatory checkpoints that can have key roles in the establishment of immunopathology and long-term disease outcomes.

## 4 RIG-I-like receptors in chikungunya virus sensing, host defense and evasion mechanisms

RIG−I–like receptors (RLRs), which include RIG-I (Retinoic acid-inducible gene I), MDA5 (Melanoma differentiation-associated protein 5) and the regulatory protein LGP2 (Laboratory of Genetics and Physiology 2), are cytosolic RNA sensors that play a central role in initiating antiviral responses against RNA viruses (**Figure 1**) (18, 81, 82). RIG-I preferentially detects short double-stranded RNA (dsRNA) segments with an uncapped 5′-triphosphate end, whereas MDA5 is more responsive to longer dsRNA molecules with structural features that do not require a 5′-triphosphate for recognition (83–86). LGP2 lacks the CARD domain and, thus, does not signal independently but modulates RIG−I (87)and MDA5 activation, facilitating viral RNA recognition by those receptors (87)or competing with them (78). Upon ligand binding, RIG−I and MDA5 undergo to conformational changes that allow them to interact with the adaptor protein MAVS (also known as Cardif/VISA/IPS-1), triggering downstream signaling cascade that lead to IFN-I production (18, 20).

RIG-I senses short 5’-triphosphate or 5’-diphosphate dsRNA. In contrast, MDA5 and LGP2 do not show preferential binding to any defined region of the CHIKV RNA (88, 89). Although LGP2 was found physically associated with CHIKV RNA during infection, there is no direct functional evidence of its involvement in the response against CHIKV. The known regulatory role of LGP2 in other viral infections suggests it may modulate RLR signaling, but its specific impact in the context of CHIKV remains to be elucidated, although its upregulation upon CHIKV infection has been demonstrated (90) (**Table 2**).

**Table 2:** RLRs in CHIKV infection ranked by functional relevance.

RLR	Expressing cell populations	Cell compartment	Ligand	Function in CHIKV infection	Reference
RIG-I	Fibroblasts	Cytosol	Short dsRNA (5′-pp or 5′-ppp)	Initiates type I IFN response.	(20)
MDA5	Fibroblasts	Cytosol	Long dsRNA	Cooperates with RIG-I to amplify IFN-I response.	(20)
LGP2	Fibroblasts	Cytosol	dsRNA	Interferes in RIG-I signaling via competitively binding to ligands? Interferes with the recruitment of MAVS? Facilitates viral RNA recognition?	(78)

RIG-I, Retinoic acid-Inducible Gene I; MDA5, Melanoma Differentiation-Associated protein 5; LGP2, Laboratory of Genetics and Physiology 2; MAVS, Mitochondrial Antiviral Signaling protein; UTR, Untranslated Region; dsRNA, Double-stranded RNA.

Experimental studies have highlighted the key role of the RLR-MAVS axis in the detection of CHIKV PAMPs and initiation of an effective antiviral response against CHIKV and other alphaviruses (91). Activation of RIG-I-like receptors by CHIKV has been shown to contribute to IPS-1/MAVS-dependent signaling and robust induction of IFN-I genes (92). An interesting study in mice showed that infected fibroblasts respond to CHIKV infection via a Cardif/MAVS-dependent sensor, producing type I IFNs (specially IFN-β) therefore limiting the infection, and the cooperation between RLR and MyD88-dependent pathways appears essential to control viral dissemination (20). While Mavs^–/–^ mice show increased viremia and systemic inflammation, they still produce high levels of systemic IFN-I (93), possibly by compensatory mechanisms through other PRR. Conversely, another study showed that serum levels of IFN-α/β was higher in wild-type (WT) mice when compared to MyD88^−/−^, TRIF^−/−^, and IPS-1/MAVS^−/−^ mice; and RIG-I signaling via IPS-1/MAVS would be the most important pathway for IFN-α/β production in response to CHIKV infection (94). However, further studies are still needed to establish the main pathway of type I IFN induction in response to CHIKV infection. Studies using bone marrow chimeric mice showed that MAVS expression in nonhematopoietic cells is essential for controlling CHIKV infection (95). In contrast, effective viral control via TLR3 depends on its expression in hematopoietic cells (56), indicating that different viral sensors have specialized functions depending on the cell type.

Comparing the MYD88, TRIF and MAVS pathway in the CHIKV susceptibility, it was shown that TRIF^−/−^, IPS-1/MAVS^−/−^, and MyD88^−/−^ mice displayed significantly higher viremias than WT. Foot swelling was significantly more pronounced in TRIF^−/−^ and IPS-1/MAVS^−/−^ mice, starting earlier, peaking higher, and lasting longer (particularly in IPS1/MAVS^−/−^ mice) than in WT mice (94). Interestingly, *in vivo* analysis indicates that RIG-I and MDA5 may have overlapping functions, since mice lacking either RIG-I or MDA5 individually did not show significant differences in CHIKV tissue loads compared to wild-type animals (20).

Further supporting the antiviral role of RIG-I, treatment of cultured fibroblasts with synthetic 5′-triphosphorylated RNA (5′pppRNA), a potent RIG-I agonist, induces a robust antiviral response and significantly inhibits CHIKV replication *in vitro*, suggesting a therapeutic potential of RIG-I agonists (96). Moreover, cell lines overexpressing RIG-I demonstrate increased resistance to viral replication (88). In human infections, elevated expression of RIG-I and MDA5 transcripts during the acute phase of CHIKV correlates with increased levels of IFN-β and IL-12, in addition to reduced viral load (58), suggesting that RLR activation contributes not only to viral clearance but also to the modulation of disease severity in natural infection.

The importance of MAVS in preventing CHIKV-induced cardiac pathology was demonstrated in MAVS-deficient mice, where CHIKV was not cleared from the heart, leading to the infiltration of CD3+ and CD11b+ cells to the tissue, prolonged myocarditis and vasculitis, despite systemic type I IFN production. In immunocompetent animals, CHIKV directly infects cardiac fibroblasts, inducing a local type I IFN response in both infected and neighboring non-infected cells, which contributes to viral clearance from the heart without causing significant tissue damage (93).

Because of the crucial role of RLR pathways in antiviral defense, CHIKV has evolved strategies to circumvent it, which can enable efficient viral replication and persistence in infected cells. It has been shown that CHIKV structural (E1 and E2) and non-structural (nsP2) proteins are capable of strongly downregulating MDA5, RIG-I, MAVS, IKKϵ, and TBK1. Also, nsP2 inhibits MDA5-induced (97) activation of the NF-κB promoter and it is a strong antagonist of IFN-β induction upon viral infection. In addition, CHIKV nsP1 also contributes to immune evasion by regulating the capping status of viral RNA. This non-structural protein functions as both capping and decapping enzymes, dynamically modulating the 5′-end of viral RNA to prevent recognition by RIG-I, which preferentially senses uncapped or 5’-triphosphorylated RNAs. By capping its RNA or removing detectable moieties, CHIKV may reduce exposure of PAMPs to host sensors (98). This mechanism is similar to the “cap-snatching” strategy used by other viruses (99), and enables CHIKV to regulate viral replication, modulate immune detection, and suppress host cell function.

In summary, the RLR pathway plays a central role in host defense against CHIKV, with MAVS emerging as an essential adaptor for the induction of IFN-I and viral clearance, especially in non-hematopoietic cells. Although RIG-I and MDA5 appear to have partially redundant functions *in vivo*, both contribute to antiviral responses and disease modulation. Moreover, cooperation between RLR signaling and MyD88-dependent TLR pathways enhances the robustness of interferon responses and viral control. Despite evidence of LGP2 binding to CHIKV RNA, its specific functional role in this context remains unclear, highlighting a promising avenue for further investigation of a possible role in the innate immune crosstalk that shapes CHIKV pathogenesis.

## 5 The role of inflammasomes in chikungunya virus infection

Inflammasomes are cytosolic multiprotein platforms composed of a sensor molecule from either the AIM2-like receptor (ALR) family or the nucleotide-binding domain and leucine-rich repeat (NLR) family, in association with the adaptor protein ASC (apoptosis-associated speck-like protein containing a CARD) and the inactive precursor enzyme procaspase-1 (100, 101). Upon sensing pathogenic or damage-associated stimuli, as potassium efflux or reactive oxygen species, procaspase-1 is cleaved into its active form, caspase-1, which subsequently processes pro-IL-1β and pro-IL-18 into their active forms IL-1β and IL-18, thereby promoting their secretion. This mechanism is central to innate immune surveillance and the orchestration of inflammation (102) and may also result in pyroptosis, an inflammatory form of apoptosis (103).

CHIKV infection has been shown to activate both NLRP3 and AIM2 inflammasomes (**Figure 1**) (90, 104), and the increase of NLRP3 transcripts expression has been reported during the acute phase (105), particularly at the peak of inflammation (104). NLRP3 activation has been associated with autophagy, pyroptosis, apoptosis of infected cells, and cytokine production in response to CHIKV infection (105–108). Indeed, the levels of IL-18 and IL-1β progressively rise as the disease progresses from the acute to the chronic phase (109), and elevated caspase-1, IL-18 and IL-1β have been detected in both patients and murine bone marrow-derived macrophages, which also release LDH (indicative of pyroptosis) upon CHIKV infection (104, 105).

Experimental models of chronic infection demonstrated that infiltrating macrophages in the joint, as well as fibroblasts harboring replicating CHIKV RNA, express increased levels of NLRP3 and IL-1β transcripts (110). Sustained NLRP3 activation by macrophages appears to be a major pathogenic driver of the transition from acute to chronic joint inflammation, primarily through persistent IL-1β induction. Importantly, pharmacological inhibition of NLRP3 using the small molecule MCC950 significantly attenuates CHIKV-induced inflammation, prevents osteoclast-driven bone loss and myositis, and decreases levels of IL-6, CCL2/MCP-1, and TNF in joint tissue, without impairing viral replication (104). Conversely, caspase-1 silencing enhances viral replication in human dermal fibroblasts, underscoring inflammasome’s possible antiviral function (90). Furthermore, hearts from CHIKV-infected Casp1/11^−/−^ mice harbored fewer infectious CHIKV particles than WT controls as early as 2 days post-infection, suggesting a role for inflammasomes in the initial stages of viral replication in cardiac tissue (93). Interestingly, platelets from CHIKV patients also exhibit increased expression of NLRP3, caspase 4 (a component of the non-canonical inflammasome pathway) and cleaved IL-1β, demonstrating that CHIKV also triggers inflammasome activation in platelets (111).

Human NLRP1 contains both an N-terminal pyrin domain (PYD) and a C-terminal caspase recruitment domain (CARD), and in keratinocytes it assembles into functional inflammasomes (112). CHIKV has been shown to activate NLRP1 (113) possibly by the detection of dsRNA (114), placing this receptor as an important sensor for CHIKV detection in the skin. Additionally, AIM2 inflammasome, traditionally described as a cytosolic DNA receptor sensing bacterial DNA (115), is also activated in CHIKV-infected dermal fibroblasts (90). However, whether AIM2 activation in this context is triggered by self-DNA, mitochondrial DNA damage (116), or direct viral PAMPs recognition remains unresolved. Interestingly, silencing AIM2 or caspase-1 expression before CHIKV infection completely abolishes IL-1β production, emphasizing the important role of AIM2 in inflammasome-driven inflammation during CHIKV infection (90).

Overall, activation of NLRP3 and AIM2 inflammasomes, possibly triggered by potassium efflux in CHIKV-infected cells, underlies disease pathology primarily through excessive and sustained production of IL-1β. Their contribution to viral control, however, remains controversial and warrants further investigation. Translating inflammasome biology into targeted therapies for CHIKD and other related arthritogenic alphaviruses represents a promising yet still largely unexplored avenue that could mitigate chronic inflammation while preserving essential antiviral immunity.

## 6 DNA sensors in CHIKV infection: cGAS and IFI16

Cyclic GMP-AMP synthase (cGAS) and its adaptor, STING (stimulator of interferon genes), initially characterized as sensors of endogenous or nonself DNA, are now increasingly recognized for their involvement in infections by RNA viruses, probably through the detection of mitochondrial DNA (mtDNA) released during virus-induced mitochondrial damage (117). Indeed, CHIKV infection has been associated with cytoplasmic accumulation of DNA in infected human cells, which can act as a DAMP and be sensed by the cGAS-STING axis (118, 119) (**Figure 1**).

Experimental studies have demonstrated that activation of cGAS-STING signaling restricts CHIKV replication (120) in both fibroblasts and immune cells (118), thereby playing a protective role. Consistently, *in vitro* CHIKV infection of murine embryonic fibroblasts derived from golden ticket mice carrying a non-functional STING variant (I199N), or of murine macrophages (RAW 264.7) deficient in cGAS or STING, resulted in exacerbated viral replication (118). *In vivo*, STING signaling has also been shown to be crucial for restricting CHIKV replication and arthritis pathogenesis, as its absence leads to higher viremia, greater joint damage, and exacerbated inflammation (119). Pharmacologic activation of STING induces a robust type I IFN response and effectively suppresses CHIKV and other alphaviruses (121, 122). Moreover, the STING agonist cAIMP provides protection in a chronic CHIKV-arthritis mouse model (123), underscoring the therapeutic potential of targeting this pathway.

To counteract these host defenses, CHIKV has evolved multiple mechanisms to antagonize cGAS-STING pathway. The viral nonstructural protein nsP1 directly interacts with STING, preventing its dimerization, thereby impairing STING activation and downstream IFN-I induction (118) while simultaneously stabilizing itself to promote viral replication, transcription, and particle assembly (124). In addition, CHIKV capsid protein cooperates with the autophagy factor ATG7 to mediate cGAS degradation via autophagy, preventing the activation of this pathway and further suppressing IFN-β production (118). Indeed, CHIKV has been shown to trigger autophagy, a process that ultimately facilitates viral replication (125).

In addition to cGAS, IFNγ-inducible protein 16 (IFI16) is another DNA sensor that contributes to STING activation and recognizes damaged DNA intracellularly (126, 127). Beyond its canonical role as a DNA sensor, IFI16 has recently been identified as a novel RNA-binding protein capable of inducing IFN-I (128), and directly interacting with CHIKV RNA to suppress viral replication and maturation (129). IFI16, which is also a member of ISGs, enhances RIG-I activation (128) and is up-regulated following CHIKV infection in human fibroblasts (130). Functional studies demonstrated that IFI16 overexpression completely abolished CHIKV replication, whereas IFI16 silencing markedly enhanced viral propagation. Moreover, IFI16 restricts viral dissemination by inhibiting both cell-to-cell transmission and extracellular spread (131). Collectively, these findings establish IFI16 as a potent intrinsic antiviral effector against CHIKV.

Overall, the current available data supports the important role of cGAS and IFI16 as key DNA sensors that can act strengthening the antiviral immune response during CHIKV infection, and the therapeutic potential of this axis is underscored by the protective effects of STING agonists in chronic CHIKV-arthritis models. However, as many other infecting pathogens, CHIKV has evolved intricate mechanisms to overcome the host defenses, as shown by the disruption of the cGAS-STING pathway, thereby ensuring its replication. Therefore, elucidating how these pathways can shape CHIKV pathogenesis, and how the virus subverts the host defense mechanisms, may represent key targets for therapeutic intervention and development of effective antiviral immunotherapies.

## 7 Mechanisms of protection mediated by innate antiviral cytokine responses during chikungunya virus infection resource identification initiative

During the acute phase of CHIKV infection, a robust innate cytokine and chemokine response is triggered, playing dual roles in host defense and disease pathology. Type I interferons, which includes IFN−α and IFN−β, have autocrine and paracrine actions and are absolutely essential to CHIKV control, driving early antiviral defense before the onset of adaptive immunity (54, 132). HeLa cells or macrophages pre-treated with either IFN-α, IFN-β, or IFN-γ and subsequently infected with CHIKV show inhibited viral protein expression (54). The absence of IFN-I signaling in Ifnar1^-^/^-^ mice, or the lack of IFN-I production in Irf3^-^/^-^Irf7^-^/^-^ double-knockout mice, leads to rapid lethality, hemorrhagic fever, and shock (94, 133). Early after infection, the rapid induction of local production of both IFN-α and IFN-β, by non-hematopoietic infected cells such as fibroblasts, but not by hematopoietic cells (cDCs and pDCs), play a central role in controlling CHIKV replication and dissemination (20). It is important to note that the induction of these antiviral cytokines is a coordinate event of multiple innate receptors and pathways, as shown by *in vivo* experiments where none of the single sensor-deficient mice are susceptible to CHIKV infection as IFNAR^−/−^ animals (20).

Despite signaling via a shared receptor (IFNAR1/2), IFN-α and IFN-β exhibit non-redundant functions in CHIKV infection: IFN-α plays a critical role in limiting persistent infection, as mice lacking IFN-α shows significantly higher viremia and viral tissue burden, and it also limits survival of cells infected by CHIKV at sites of dissemination, particularly dermal fibroblasts and immune cells, thereby acting early to control chronic CHIKV pathogenesis. In contrast, IFN-β contributes to modulating neutrophil-mediated inflammation and tissue damage in the acute phase of the disease (134, 135) (**Table 3**). Mice deficient in IFN-β had more severe joint swelling but decreased IL-1β, TNF-α, CXCL9/MIG, CXCL10/IP-10, CCL2/MCP-1, CCL3/MIP-1α, CCL5/RANTES, and eotaxin despite relatively normal viral control (134).

**Table 3:** Differences and similarities between IFN-α and IFN-β in CHIKV Infection.

Feature	IFN−α	IFN−β	Reference
Main source	Nonhematopoietic cells (Fibroblasts).	Nonhematopoietic cells (Fibroblasts).	(20)
Control of viremia	Limits early viral replication and dissemination.	Dispensable for controlling viral burden.	(20, 134, 139)
Target	Strongly detected in the serum and in PBMC transcripts of patients during the acute phase.	Detected in PBMCs from patients during the acute phase, with a prominent role in tissues (*e.g.*, musculoskeletal tissues and heart).	(20, 58, 205)
Modulation of inflammation	Limited role.	Controls cellular infiltration into the midfoot joint spaces; Limits neutrophil-mediated inflammation; Modulates inflammatory mediators.	(134)
Prevention of chronic symptoms	Essential to prevent viral persistence and chronic pathology.	Not sufficient to prevent chronic disease.	(135)
Effect ISGs	Strong inducer of several ISGs.		
Effect of cytokine modulation	Inhibited by IL−17A	Positively modulate IL-1β, TNF-α, CXCL9, CXCL10, CCL2, CCL3, CCL5, and eotaxin at 2.	(134, 206)
	IRF3/7^−/−^ ↑IFN-γ and TNF.		(94)
Viral evasion		Antagonized by E1 and E2 proteins.	(155)
	Suppressed by nsP2-mediated inhibition of JAK-STAT signaling downstream of IFNAR.		(44)
Clinical relevance	Prophylactic and therapeutic administration prevents mortality; Pretreatment prevent arthritis; + Favipiravir suppresses viral burden *in vitro*; + Ribavirin (?)	Pretreatment inhibits RNA translation.	(136–138, 140)

Given its strong antiviral properties, several studies have investigated the prophylactic and therapeutic potential of type I IFN against CHIKV. In murine models, a single administration of the adenovirus-vectored murine IFN-α (mDEF201) protects against a lethal challenge of CHIKV, with protective effects observed even when treatment was initiated post-infection (136).

The antiviral potential of IFN-α has also been evaluated in combination regimens: IFN-α and favipiravir exhibited synergistic inhibition of CHIKV replication in human connective tissue (HT-1080) and neuronal (SK-N-MC) cell lines, but not in skin fibroblasts (HFF-1). Notably, in monotherapy, IFN-α displayed the highest antiviral potency in HFF-1 cells, followed by HT-1080, and then SK-N-MC, in a dose-dependent manner (137). Regarding ribavirin, strong synergy between ribavirin and IFN-α2a in CHIKV-infected Vero cells was reported (138), whereas no significant benefit and noted substantial cytotoxicity in human cell models was found (137), highlighting the need for further investigation. In addition, prophylactic IFN-α administration in the context of CHIKV infection was able to prevent arthritis (139), and prophylactic IFN-β treatment protected against CHIKV infection by inhibiting translation of incoming viral mRNAs through both IFIT1-dependent and IFIT1-independent mechanisms, without affecting viral entry or disassembly (140) (**Table 3**).

Experimental findings have shown that the IFN-I response limits viral replication by orchestrating the expression of hundreds of SGs, which collectively establish an antiviral state within host cells. Notably, the antiviral activity of ISGs is virus-specific. Using an overexpression screening approach in human fibroblasts followed by viral infection, it was demonstrated that several ISGs effectively inhibit CHIKV replication, and the ISGs P2RY6, SLC15A3, and SLC25A28 displayed CHIKV-specific antiviral activity not observed for other viruses tested. Conversely, the double-stranded RNA-specific adenosine deaminase (ADAR) acts as a proviral factor, enhancing CHIKV replication (141). These results prompted further functional screening of ISGs to clarify their roles in CHIKV infection and pathogenesis. Furthermore, research using Stable Isotope Labelling by Amino acids in Cell culture (SILAC)-based mass-spectrometry analysis in CHIKV-infected human skin fibroblasts showed an upregulation of the ISGs MX1, IFIT1, IFIT3, and ISG15 (142). Among these, the ISG15, typically known for its antiviral role through conjugation to target viral proteins, emerges as a critical effector in controlling CHIKV infection in a non-classical route independently of the conjugation. Neonatal mice lacking ISG15 exhibit dramatically increased mortality and higher levels of inflammatory mediators upon CHIKV infection (143).

Another ISG, viperin, is transcriptionally induced during CHIKV infection in PBMCs, while its antiviral activity has been demonstrated in different experimental models (144). Furthermore, viperin-deficient mice infected with CHIKV exhibit an exacerbated Th1-skewed immune response, elevated IFN-γ levels in the joint microenvironment, and enhanced infiltration of inflammatory monocytes into infected joints, when compared to their wild-type counterparts (145). Antiviral genes for OAS1 (2’,5’-oligoadenylate synthetase 1) and PKR (protein kinase activated by dsRNA) expression were also found to be upregulated in human monocytes and in MDMs after CHIKV infection (62).

Interferon-induced transmembrane proteins (IFITMs), particularly IFITM1, IFITM2, and IFITM3, are a class of broadly acting ISGs known to restrict a variety of RNA viruses, including CHIKV (142). These small membrane-associated proteins function primarily by altering the properties of host cell membranes, thereby restricting viral entry and fusion events. IFITM3, restricts CHIKV entry and fusion in a pH-dependent manner. Its *in vitro* depletion significantly enhanced CHIKV replication in human fibroblasts, and Ifitm3^−/−^ mice infected with CHIKV displayed elevated viral loads in the spleen, serum, and joint tissues during the early stages of infection compared to wild-type animals. IFITM1, 2, and 3 also restrict CHIKV infection at the level of glycoprotein-mediated entry (146). In accordance, the overexpression of IFITM1, 2, or 3 in human skin fibroblasts robustly inhibits CHIKV replication, while knockdown of these genes increases the viral load (142). Interestingly, the antiviral activity of IFITMs was associated with increased transcription of TLR3, TLR7, TLR8, TLR9, downstream signaling components TRADD, IRAK1, TRAF6, and MAP3K7, IRF 3/5/7, and reduced expression of LGP2 (78). Together, these findings position IFITM proteins as critical effectors of innate antiviral immunity against CHIKV, acting not only by directly restricting virus entry, but also modulating innate signaling pathways and immune cell activation. Future studies dissecting the specific mechanisms of IFITM-mediated restriction and their interplay with PRR pathways in different cell types will be essential for fully elucidating their role in CHIKV pathogenesis and potential therapeutic targeting.

The ISG IFIT1 plays a critical role in the IFNβ-mediated inhibition of CHIKV RNA translation; however, an IFIT1-independent mechanism has also been demonstrated to contribute to this antiviral effect (140). ISG20 is a 20-kDa ISG product that functions as a 3′–5′ exonuclease, and is located in the Cajal bodies in the nucleus. ISG20 modulates the production of IFIT1 and so it has an indirect role in the early restriction of RNA CHIKV replication (147). Nucleophosmin 1 (NPM1/B23), a nucleolar phosphoprotein, also restricts CHIKV by modulating IFIT1 and OAS3 (148). Overexpression of OAS3 in human epithelial HeLa cell lines was shown to inhibit early stages of CHIKV replication, while HeLa cells expressing a truncated variant of OAS3 showed reduced resistance to CHIKV infection (149). This suggests that OAS3 polymorphism in humans may be involved with susceptibility to Chikungunya fever.

The ISG known as bone marrow stromal antigen 2 (BST-2), also called Tetherin, functions as a membrane-associated restriction factor induced by type I IFN, efficiently inhibits the release of CHIKV particles from host cells without affecting virus entry and infection (150). Studies using virus-like particles (VLPs) have demonstrated that BST-2 robustly retains budding CHIKV VLPs at the plasma membrane, thereby hindering viral release and preventing the infection of bystander cells. BST-2 co-localizes with the CHIKV E1 glycoprotein on the cell membrane. A study using mouse models corroborate the antiviral significance of BST-2 in CHIKV infection, as BST-2–deficient mice exhibit heightened systemic viral loads, increased lymphoid tissues tropism, and a reduction in key antiviral factors upon CHIKV challenge, underscoring BST-2’s role in restricting virus dissemination *in vivo* (151). Additionally, the ISG product TMEM45B, a transmembrane protein that was detected mainly in the trans-Golgi network, endosomes, and lysosomes, plays a role in inhibiting the translation and promoting RNA degradation. TMEM45B interacts with Nsp1 and Nsp4 of CHIKV, but its direct role in inhibiting CHIKV replication still needs to be evaluated (152).

In a model of CHIKV neurovirulence, it was demonstrated that several ISGs have increased transcript levels in both in mouse brain and human neuronal cells line (IMR-32), including Ifit1, Ifi44, Ddx60, Usp18, and Mnda genes (153). The East Central South African (ECSA) genotype of CHIKV with E1:A226V mutation was shown to be related to unusual severe manifestations, including neurological disorders. It was shown that this mutant induces lower levels of antiviral genes IFN-β, OAS-3, MX-2, ISG-15, TLR3 and 7 as compared to non-mutant strain in neuroblastoma cell line (N2a) (19).

Despite the antiviral potency of IFN-I, CHIKV has also evolved multiple strategies to evade this response. It was shown that the viral non-structural protein nsP1 antagonizes BST-2 by downregulating its expression, which rescues viral particles release (154). Notably, the nsP2 can inhibit JAK-STAT signaling downstream of the IFNAR receptor, suppressing the expression of ISGs and facilitating viral persistence (44). In addition, the structural proteins E1 and E2 also contribute to immune evasion by antagonizing the IFN-β signaling pathway, further impairing the host’s antiviral response (155). Another sophisticated immune evasion strategy employed by CHIKV is through the C-terminal methyltransferase-like domain of nsP2, promoting the nuclear export of phosphorylated STAT1, thus preventing proper induction of ISGs (156). Although CHIKV stimulates the transcription of classical antiviral ISGs, studies in infected human fibroblasts indicate that the production of some corresponding proteins, such as ISG56 and viperin, may be suppressed at the translational level (92), highlighting a viral strategy to uncouple ISG transcription from effector protein expression.

Finally, interferon lambda (IFN-λ), a type III IFN also known as IL-28/29, is capable of inducing antiviral response both *in vitro* and *in vivo* (157, 158). However, its role in CHIKV infection has weak evidence. *In vitro* data showed that the IFN-λ pretreatment of vero cells limited viral growth, whereas its administration after infection enhanced CHIKV replication (159). Further studies are therefore needed to clarify the *in vivo* role of IFN-λ during CHIKV infection. In contrast, IFN-γ is elevated in recovered individuals compared to acute patients, and a negative correlation between IFN-γ levels and CHIKV load is observed in acute patients, suggesting its antiviral role (160). Another cytokine with important antiviral activity in the context of CHIKV infection is IL-27. In human MDMs, CHIKV induces high levels of IL-27, and similar to IFNs, IL-27-mediated response led to reduced CHIKV replication in these cells in a dose-dependent manner (74, 161). Interestingly, human THP-1-derived macrophages treated with rIL-27 upregulates the expression of several ISGs, including viperin, ISG20, IFIT1,2,3, PKR, OAS1,2,3, and others (74). Comparative transcriptomic analysis further revealed that IL-27 is able to activate many of the same antiviral and inflammatory pathways triggered by IFN-α, IFN-γ, and IFN-λ in MDMs, including those involved in JAK-STAT signaling. On this basis, IL-27 has been proposed as a novel member of the IFN family, called IFN-π, the type V IFN (161). IL-27 has also been suggested (162) as a chronicity biomarker and may also contribute to CHIKD pathogenesis. Elevated serum levels of IL-27 have been observed in patients in the chronic phase of CHIKD (>12 weeks) compared with those in subacute phase (3–12 weeks), correlating with joint tenderness, and suggesting a dual role for IL-27 in both antiviral defense and immunopathology (162).

## 8 Innate cytokines and chemokines-mediated immunopathology during CHIKV infection

Innate cytokines play a pivotal role in shaping the host immune response to infections, acting as early mediators that link pathogen sensing with the activation of antiviral and/or inflammatory pathways. A hallmark of CHIKD is the robust induction of inflammatory cytokines. While IFNs-I are essential for controlling viral replication (20), excessive or dysregulated production of pro-inflammatory mediators, including IL-6, IL-1β, TNF-a, CCL2/MCP1, CXCL10/IL10, during acute and/or chronic phases has been implicated in joint swelling, persistent arthralgia, neurological involvement, and other sequelae (**Table 4**). Understanding the timing, sources and regulation of these responses is critical to unraveling CHIKV immunopathogenesis and for identifying therapeutic targets and effective vaccines.

**Table 4:** Innate cytokines and chemokines involved in human CHIKD pathogenesis.

Mediator	Acute phase	Chronic phase	Convalescent or fully recovered	References
IL-6	↑ Correlated with fever, high viremia, cutaneous rash, joint pain and lethal case.	↑ Associated with musculoskeletal pathology, persistent arthralgia, neurological involvement (also present in CFS).	↑ In patients with persistent arthralgia ↓ Fully recovered	(58, 160, 163–172, 181, 182, 196, 202)
IL-1β	↑ Correlated with disease severity and high viral load.	↑ Linked to persistent arthralgia	↑ In patients with persistent arthralgia	(160, 164, 166–172, 181, 202)
TNF-α	↑ Associated with skin manifestations	↑ Neurological involvement (also present in CFS).	Conflicting data	(164, 169, 196, 202)
IL-8/ CXCL8	Conflicting data	Conflicting data	Conflicting data	(160, 164, 168–172, 177, 202)
GM-CSF	↑	↑ Linked to persistent arthralgia	↓	(160, 166, 173)
CCL2/ MCP-1	↑ Associated with monocyte recruitment, limit neutrophil-mediated pathology; Linked to greater disease severity and higher viral load	↑ In patients with persistent arthralgia; Neurological involvement (also present in CFS).	↑ In patients with persistent arthralgia ↓ Fully recovered	(23, 164, 169–172, 181, 182, 202)
CXCL9/MIG	↑ Associated with greater disease severity and higher viral load	↑ Neurological involvement (Present in CFS).	↓ Fully recovered	(164, 170–172, 181, 182, 202)
CXCL10/IP-10	↑ Associated with greater disease severity	↑	↑ In patients with persistent arthralgia ↓ Fully recovered	(164, 170–172, 181, 182)
CCL5/ RANTES	Conflicting data	↓ In serum in severe pain ↑ In CFS of neurological patients	?	(169–172, 174, 196, 202)
CCL4/ MIP-1b	↑	↑ In patients with persistent arthralgia	?	(174)
IL-12	↑	↑ Persistent arthralgia and severe pain	↑	(58, 164, 169–172)
IL-18	↑	↑	↑	(175, 176)
IL-10	↑	?	Conflicting data	(164, 170–172, 207)
IL-5	?	?	↑	(164)
IL-4 and IL-13	↓	?	?	(176)
Eotaxin & HGF	?	?	↑	(166)

↑, increase expression; ↓, decrease expression;?, no data available.

IL, interleukin; TNF, tumor necrosis factor; HGF, hepatocyte growth factor; GM-CSF, granulocyte-macrophage colony-stimulating factor; CCL, C-C motif chemokine ligand; CXCL, C-X-C motif chemokine ligand; MIP-1B, macrophage inflammatory protein 1 beta; MIG, monokine induced by gamma interferon; IP-10, interferon gamma-induced protein 10; RANTES, regulated upon activation, normal T cell expressed and secreted; MCP-1, Monocyte Chemoattractant Protein-1.

Over the years, multiple clinical and experimental studies have explored the contribution of inflammatory cytokines on CHIKD pathogenesis and progression, although some discrepancies remain and the precise cellular origins of these mediators are not fully defined. Among them, IL-6 has emerged as a key cytokine associated with immunopathology and clinical outcomes. Elevated IL-6 levels are frequently detected during the acute phase (58, 163, 164), often correlated with higher viral loads (163), and also with lethal disease (165). Increased IL-6 and IL-1β have been repeatedly linked to disease severity, including higher fever, viremia, and exacerbated joint manifestations (164, 166–168). Accordingly, both cytokines have been proposed as biomarkers of CHIKD severity alongside low levels of CCL5/RANTES and IL-8/CXCL8 (160, 167–169). However, larger cohorts are still required to confirm their prognostic value.

Patients with acute CHIKV infection display increased levels of IL-8/CXCL8, CCL2/MCP1, CXCL9/MIG, CCL5/RANTES, CXCL10/IP10, IL-1β, IL-6, IL-12, IL-10, TNF-α, and GM-CSF compared to healthy controls (170–173). However, another study also found the production of IL-8/CXCL8, CCL2/MCP-1, IL-6, MIP-1α, and MIP-1β to be increased during chronic phase (171), while CCL5 was decreased during acute phase (174). During the convalescence period, TNF-α, IL-6, IL-13, and CXCL-10/IP-10 levels remained elevated compared with controls (172).

Elevated levels of IL-18 (175) and reduced levels of IL-4 and IL-13 were observed in patients during the acute phase with joint pain compared to those without this symptom (176). Importantly, IL-18 levels increased significantly from day 1 to days 5–9 after symptoms onset (176), remaining even higher during convalescence than in the acute phase. The IL-18 binding protein (IL-18BP), a natural regulator of IL-18, is also elevated during acute phase but it decreases as the infection progresses to convalescence, thereby reducing its neutralization ability (175). These findings highlight IL-18 as a potential target for immunomodulation.

Contradicting results for IL-8/CXCL8 have been reported. Research showed reduced levels in acute severe cases (168), whereas others reported elevated levels during early acute infection (~3 days to 1 week after symptoms onset) (163, 164). A transient rise (1–3 days after symptoms onset) followed by a sharp decline at day 4 has also been documented (170). In contrast, other studies found no significant differences across acute, chronic, and recovered groups (160) or between patients with post-CHIKV chronic rheumatism and controls without rheumatic manifestations (177). Importantly, when categorizing the patients according to arthralgia duration, patients whose arthralgia lasted > 3 months had increased IL-8/CXCL8 levels compared to those reporting arthralgia for ≤ 3 months (170). A study designed to compare CHIKD patients with persistent arthralgia (5 years after the symptoms onset) to healthy controls, showed high levels of IL-8/CXCL8, IL-6, IL-1β, CCL2/MCP1, MMP-1, and MMP-3 in persistent arthralgia group, pointing to the role of these inflammatory mediators in chronic musculoskeletal pathology (169). Follow-up studies focusing on IL-8/CXCL8 expression and its consequences during acute and chronic phases, including both severe and non-severe disease, and patients with persistent arthralgia versus full recovery, could help to address the inconsistency within the available data and fulfill this knowledge gap.

Considering patients with persistent arthralgia during the chronic phase 2–3 months after illness onset, elevated IL-6 and GM-CSF were detected when compared to those patients who had fully recovered (166). In contrast, fully recovered patients exhibited increased levels of eotaxin and hepatocyte growth factor (166), in addition to decreased levels of TNF-α, MMP-1, and MMP-3 than patients with persistent arthralgia (169). When considering only patients with persistent arthralgia, high levels of TNF-α, IL-12, and CCL2/MCP1, and low levels of CCL5/RANTES were detected in those with severe pain compared to the non-severe pain group (169). In contrast, low TNF-α levels during the acute phase were predictive of chronic joint pain, suggesting a dual and stage-specific role of TNF-α in CHIKV pathogenesis (178), as observed for IL-8/CXCL8. The treatment with anti-TNF-α has been proposed for chronic chikungunya induced arthritis (179), as in patients with chronic inflammatory arthropathy using anti-TNF drugs, who were also CHIKV positive, did not develop severe manifestations during the acute stage and exhibited a reduced occurrence of persistent joint symptoms compared to household control (no diagnosis of inflammatory disease or use of immunosuppressants and CHIKV+) (180).

In a cross-sectional study involving 196 acute CHIKD patients in India, elevated levels of CXCL10/IP-10, CCL2/MCP-1, and CXCL9/MIG were associated with greater disease severity. In the convalescent phase (1 month post-symptom onset), IL-6, IL-1β, IL-9, and CXCL10/IP-10 production remained elevated in patients with persistent arthralgia, whereas IL-4 and IL-10 declined (181). Another study from the 2007 Italian outbreak, tracked the serum levels of 13 cytokines and chemokines across three time points: acute phase, and follow-up at six and twelve months. During the acute stage, levels of IL-6, CXCL9/MIG, CCL2/MCP-1, and CXCL10/IP-10 were significantly elevated compared to later time points (acute vs. 6- or 12-month follow-up), when they declined. Over time, there was a marked increase in the levels of IL-1β, TNF-α, IL-12, IL-10, IFN-γ, and IL-5, indicating a prolonged or evolving immune response during the convalescence phase of CHIKV infection. Additionally, higher levels of CXCL9/MIG and CXCL10/IP10 were detected in the very early acute phase (164), and their decrease during convalescence associates, together with declining CCL2/MCP1, with disease severity (182). Additionally, CXCL9/MIG and CCL2/MCP-1 were higher in acute patients with higher viral load compared with those with low viral loads (163).

Additional insights from mechanistic studies, showed the role of CXCL10/IP10 signaling in promoting viral persistence, leukocyte trafficking, and inflammation during CHIKV infection. It was demonstrated, using a Cxcl10^−/−^ mice model, that CXCL10/IP10 deficiency led to lower viral loads in the infected ankle joints at days 4 and 7 post infection, reduced numbers of immune cells recruited to the muscles and joints, and lower expression of IL-6, TNF-α, and IFN-β1 mRNA transcripts in joints compared to WT (183). Notably, treatment with atorvastatin, a clinically approved cholesterol-lowering drug that suppresses CXCL10 expression in mice and humans, was able to reduce CHIKV-driven inflammation and pathology during the acute phase, positioning the CXCL10–CXCR3 axis as a potential therapeutic target for CHIKV-associated arthritis (183).

The induction of pro-inflammatory cytokines also seems to be strain-dependent. In murine models, the Réunion Island isolate from the 2005 outbreak (184) induced markedly higher serum levels of CCL2/MCP-1, IFN-γ, and IFN-α/β during viremia, and increased TNF-α and CCL2/MCP1 during the symptomatic phase, compared to an Asian-lineage 1960s isolate. The Réunion Island isolate also drove more severe rheumatic disease (139).

*In vitro*, CHIKV infection in monocytic U937 cell line upregulates the expression of IL-1β, IL-6, IFN-γ, and TNF-α mRNA transcripts, reinforcing a myeloid origin for these inflammatory mediators in human infection (185). In non-human primates, the Réunion Island isolate led to increased systemic levels of IFN-α/β, IFN-γ, CCL2/MCP1, CCL3/MIP-1α and CCL4/MIP-1β, IL-6, and TNF-α (24), further validating the cytokine storm. In humans, CCL4/MIP-1β levels were found elevated in the chronic phase, and it is associated with arthritic symptoms (174). Although CCL2/MCP1 does not directly impact viral replication (186), a dual role of CCL2/MCP1-CCR2 axis in CHIKV-mediated disease has been described: CCL2/MCP1-CCR2 besides contributing to monocyte recruitment and inflammation, it is critical for limiting excessive pathology mediated by neutrophils (23). An overproduction of mediators regulating immune cell recruitment and activation, such as IL-8/CXCL8, CXCL10/IP10, CXCL9/MIG, and CCL2/MCP-1 may contribute to the pathological manifestations of CHIKV infection.

Meta-analysis further highlighted the complexity of the cytokine signature. One report identified a broad acute-phase profile characterized by elevated levels of IFN-α, IFN-γ, IL-2R, IL-6, IL-7, IL-8/CXCL8, IL-1Ra, IL-4, CCL2/MCP-1, CXCL9/MIG, CXCL10/IP-10, VEGF, and G-CSF (187). Another meta-analysis study associated high levels of IL-6, CXCL10/IP-10, IL-1b, CXCL9/MIG, CCL2/MCP-1, along with reduced CCL5/RANTES and CXCL8/IL-8 with CHIKD severity. In addition, chronification was marked by increased levels of IL-6, TNF-α, CCL2/MCP-1, IL-12, INF-α, IL-13, INF-γ, GM-CSF, CRP, IL-1a, IL-15, Factor VII, CXCL10/IP-10, IL-10, IL-4, IL-1RA, IL-8, MIP-1α, MIP-1β, ferritin, MIG, ESR, NO, malondialdehyde, and reduced levels of CCL5/RANTES, ferritin, eotaxin, HGF, IL-27, IL-17A, IL-29, TGF-β, IL-10, and thiols (188).

The cytokine storm observed in CHIKD is accompanied by complement activation, with elevated C3a, C5a and sC5b-9 detected during the acute phase (109, 164). In chronic patients, C3a, C5a, and C1q-binding IgG–containing circulating immune complexes (CIC-C1q) remain elevated; in recovered individuals, C3a, C5a, sC5b-9, and CIC-C1q reach their highest levels, indicating a constant activation of the complement system after CHIKV infection (109). Moreover, increased mannose binding lectin (MBL) during the acute phase and higher C3a concentrations in patients with musculoskeletal complications or severe disease further support the contribution of complement to joint-specific pathology (189). Despite this activation and deposition of complement proteins, there is evidence that CHIKV displays significant resistance to complement activity. Indeed, CHIKV exhibits a factor I-like activity that cleaves C3b into inactive C3b (iC3b), allowing the virus to evade complement-mediated virolysis and potentially contributing to alphavirus-induced arthritic symptoms (190). Furthermore, fatal outcomes of CHIKD have been associated with a dysregulated immune response that includes the downregulation of several complement proteins (C3, C4A, C4B, C1QC, C6, C8A, and C8B) (191).

Beyond the systemic response, musculoskeletal tissues represent critical sites of cytokine-driven pathology during CHIKV infection. Local stromal and structural cells, including fibroblasts, muscle satellite cells, and osteoblasts, are not only permissive to infection (192) but also actively contribute to the inflammatory milieu (193). *In vitro* and *ex vivo* experiments demonstrated that human osteoblasts secrete high levels of IL-6, RANKL, and Osteoprotegerin (the latter two involved in bone resorption and bone metabolism) following CHIKV infection (193). CHIKV can infect muscle cells, inducing degenerative changes, atrophy, necrosis, and the production of IL-6, CCL2/MCP-1, CCL5/RANTES, and TNF-α by these cells (194). Using Irf1-/- mice model infected with CHIKV, it was shown the role of this molecule in protecting muscle tissues from infection and controlling inflammation by modulating IL-4, IL-6, CXCL1, CXCL2, CCL3, and CCL4 in the joint, in addition to neutrophil and eosinophil recruitment (195). Furthermore, considering that 20-80% of patients present skin manifestations, cutaneous inflammation is also a relevant target of investigation. TNF-α, IL-6, CCL5/RANTES are expressed in the epidermis, blood vessels, sweat glands, and infiltrating cells upon CHIKV infection (196). Collectively, these findings highlight resident tissue cells as a significant source of inflammatory mediators that, together with infiltrating leukocytes, drive cutaneous rash, joint pathology, chronic arthralgia, and bone remodeling defects in CHIKD. Thus, therapeutic strategies targeting these mediators (rather than specific cellular subsets) may therefore be proved effective.

Nevertheless, infants with *in utero* exposure to maternal CHIKV infection also have an exacerbated inflammatory environment, with elevated levels of TNFα, IL-6, IL12p70, IFN-g, IL-7, IL-17A, G-CSF, VEGF, IL-1ra, IL-4, IL-10, MIP-1α, MIP-1β, IL-8/CXCL8. In addition, lower levels of PDGF-BB, GM-CSF, CXCL-10, and eotaxin were detected in those infants. One infant displayed neurological abnormalities; however, the long-term consequences of this inflammatory signature remain to be defined (197). Moreover, neurological involvement has been documented in severe CHIKV, including encephalitis, myelopathy, peripheral neuropathy, myeloneuropathy, and myopathy (198). These complications typically arise during the acute phase and can be fatal (199–201). Increased levels of TNF-α, IL-6, IL-8/CXCL8, IFN-α, CCL2/MCP1, CXCL9/MIG, CCL5/RANTES, and CCL17/TARC have been detected in cerebrospinal fluid (CSF) from CHIKD patients with neurological complications (202). Surprisingly, the levels of IL-6 and IL-8/CXCL8 in CFS of these patients were even higher than in the serum, underscoring local production of inflammatory cytokines within the CNS (202).

In summary, evidence from multiple studies indicates that a sustained inflammatory storm is central to CHIKD immunopathogenesis, orchestrating joint pathology, chronic arthralgia, and neurological complications. The dynamic regulation of these mediators, alongside the contributions of resident stromal and immune cells, underscores the multisystemic nature of CHIKD. Among these mediators, IL-6, GM-CSF, CCL2, CXCL9, and CXCL10 are consistent indicators of disease severity, remaining elevated throughout both the acute and chronic phases and declining only in fully recovered individuals, highlighting their potential as biomarkers of disease progression and chronicity. By contrast, conflicting or limited data regarding IL-8/CXCL8, TNF-a, CCL5, IL-10, IL-5, eotaxin, and HGF emphasize the need for further studies to clarify their roles and kinetics (**Table 4**). Collectively, these insights point toward therapeutic strategies targeting pathological cytokines as promising options to alleviate chronic sequelae while minimizing excessive tissue damage and preserving antiviral immunity.

## 9 Conclusion and future directions

CHIKV infection highlights the fine balance between antiviral immunity and detrimental inflammation. Innate immunity plays a pivotal role in driving the course of CHIKV infection, acting as both first line of defense against viral dissemination and as a key player for the inflammation that underlies the pathogenesis of CHIKD. The viral sensing through several pattern recognition receptors, such as Toll-like receptors and RIG-I-like receptors triggers coordinated antiviral pathways, such as robust IFN-I responses, induction of ISGs, and the release of inflammatory cytokines and chemokines. These mechanisms can restrict viral replication but also drive immunopathogenesis when its activation is excessive, prolonged, or dysregulated.

Herein, we summarized the role of TLRs, such as TLR3, TLR4, and TLR7/8, in contributing to the sensing of viral PAMPs, with distinct outcomes ranging from viral clearance to inflammatory exacerbation. However, other members of the TLR family are still underexplored in the context of CHIKV infection. Similarly, the RLR-MAVS axis has been shown to play a central role in the induction of IFN-I and contributing to viral control. On the other hand, the modulatory activity of LGP2 during CHIKV infection remains unclear despite its interaction with the viral RNA. Another important point to be taken into consideration is the role of inflammasome activation during CHIKV infection, and the robust IL-1β and IL-18 responses that are triggered by this pathway, linking acute infection with chronic joint inflammation; yet their potential antiviral contributions particularly in tissue-specific contexts are still debated.

The dual nature of innate immunity, from protective to pathogenic, defines the immunological paradox of CHIKV. Type I interferons remain indispensable for controlling acute viremia, with IFN-α and IFN-β displaying non-redundant roles in limiting persistent infection and modulating inflammation. Additionally, cytokines such as IFN-γ and IL-27 reinforce the antiviral defense but can also contribute to disease chronicity, highlighting their double-edged roles. Importantly, several inflammatory mediators, such as IL-6, IL-1β, TNF-α, CCL2, CCL2/MCP-1, CXCL9, and CXCL10 have also emerged as correlates of severity and chronicity, while conflicting evidence underlies the role displayed by IL-8/CXCL8 during CHIKV infection. On this basis, it becomes clear that sustained or dysregulated inflammatory responses can drive immunopathology, which contributes to the development of persistent arthralgia, joint damage, and even neurological complications. Understanding these dynamics is of major importance not only for elucidating the disease mechanisms, but also for the development of effective immunomodulatory antiviral therapies.

Our suggestion is that future research must focus on clarifying the precise molecular ligands and receptor interactions that initiate sensing, as well as the temporal and tissue-specific dynamics of innate signaling. The selective blockade of inflammatory cytokines (e.g., IL-6 and IL-1β) or even the modulation of inflammasome and/or TLR4-driven inflammatory pathways could help to counteract the immunopathogenesis. Additionally, identifying host genetic variants associated with TLR or other PRRs signaling may provide predictive biomarkers for disease susceptibility and severity. Conversely, the pharmacological activation of protective pathways such as cGAS-STING could contribute to enhancing viral clearance, however further research should take into consideration the viral evasion mechanisms.

Ultimately, the challenge lies in leveraging innate immunity to design interventions that achieve robust viral clearance without tipping into chronic immunopathology. By shedding light into the understanding of the complex dynamics of innate immune pathways, by combining precision-targeted therapies, could help shift the balance towards protective immunity while alleviating long-term sequelae. Nonetheless, further studies integrating mechanistic approaches, larger clinical cohorts, and therapeutic trials would be essential to turn innate immunity from a double-edged sword into a foundation for the development of effective antivirals, and immunomodulatory therapies against CHIKV and related alphaviruses.
